# Supplementary material for: Phagosomal removal of fungal melanin reprograms macrophage metabolism to promote antifungal immunity
Source: Nat Commun. 2020 May 8;11:2282. doi: 10.1038/s41467-020-16120-z (PMC7210971; doi:10.1038/s41467-020-16120-z)
Supplement: Supplementary file 1 — Supplementary Information [file 41467_2020_16120_MOESM1_ESM.pdf]

Supplementary Information

Phagosomal removal of fungal melanin reprograms macrophage metabolism to promote antifungal immunity

Gonçalves et al.

Supplementary Fig. 1

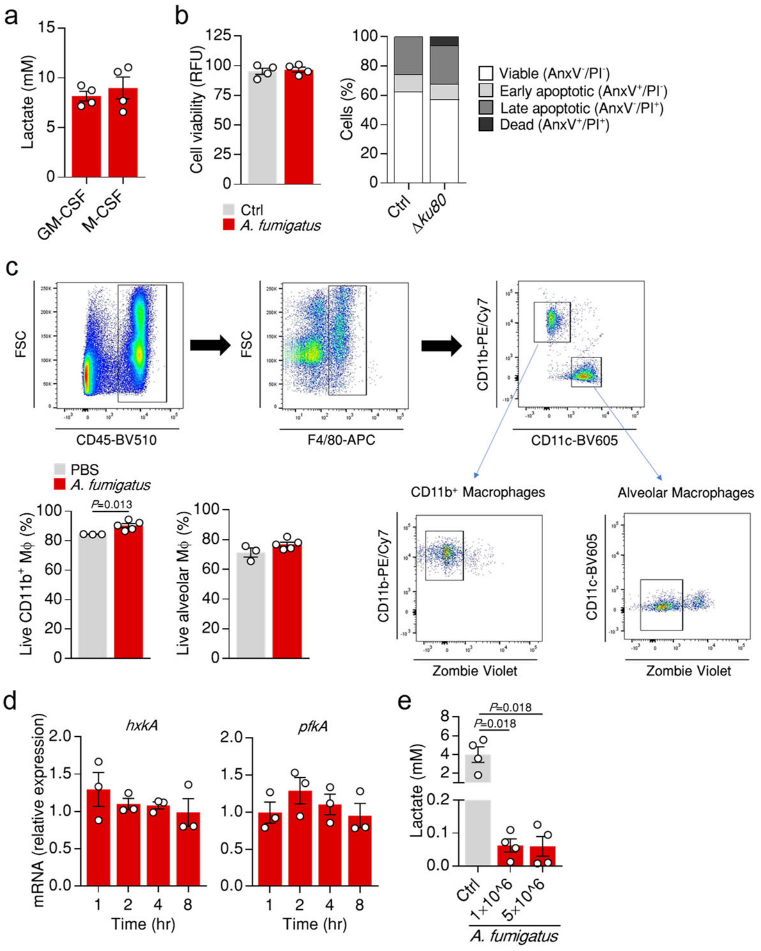

Supplementary Fig. 1 | *A. fumigatus* induces the metabolic shift of macrophages during infection. (a) Lactate secretion by macrophages differentiated from monocytes using GM-CSF or M-CSF and infected with *A. fumigatus* for 24 hr (n=4). (b) Cell viability of macrophages left untreated (Ctrl) or infected for 24 hr and determined using AlamarBlue, expressed as relative fluorescence units (RFU) (n=4), or using annexin-V/PI staining, expressed as percentage (%) of cells (n=2). (c) Flow cytometry analysis of lung cells from C57BL/6 mice after 1 day of infection with *A. fumigatus* or following a mock challenge (PBS). Leukocytes were gated by size using forward and side scatter and then on live single cells (by propidium iodide exclusion). The pan-leukocyte marker CD45 identified leukocytes, whereas macrophages were further defined as F4/80$^+$CD11b$^+$CD11c$^-$ (plots are representative of three independent experiments). Viability was determined using the Zombie Violet dye. Results are expressed as the percentage (%) of viable cells. (d) mRNA expression of *hxkA* and *pfkA* from *A. fumigatus* during infection of macrophages for 1, 2, 4 or 8 hr relative to the fungus alone (n=3). (e) Lactate secretion by macrophages left untreated (Ctrl) or by conidia of *A. fumigatus* alone cultured in vitro at two different concentrations for 24 hr (n=4). Data are expressed as mean values ± SEM; P values were calculated by Student's two-tailed t test.

Supplementary Fig. 2

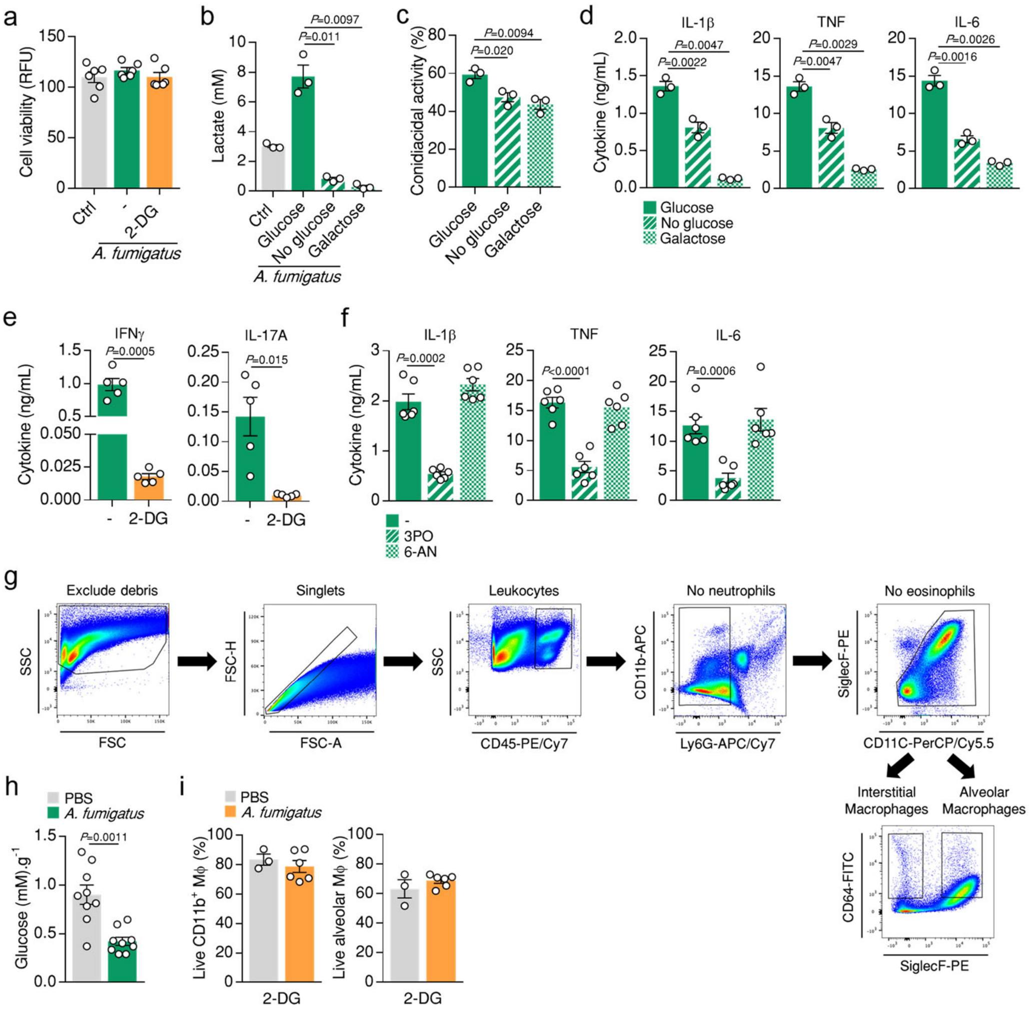

Supplementary Fig. 2 | Glycolysis is required for immune responses to *A. fumigatus*. (a) Cell viability (relative fluorescence units, RFU) of macrophages left untreated (Ctrl) or infected with *A. fumigatus* for 24 hr without or with 10 mM 2-DG (n=6). (b) Lactate secretion by macrophages either left untreated (Ctrl) or infected for 24 hr in the presence or absence of glucose or using galactose-supplemented media (n=3). (c) Conidiacidal activity and (d) production of IL-1β, TNF and IL-6 by macrophages infected for 3 or 24 hr, respectively, in the presence or absence of glucose or using galactose-supplemented media (n=3). (e) Production of IFNγ and IL-17A by PBMCs stimulated with inactivated conidia for 7 days without or with 10 mM 2-DG (n=5). (f) Production of IL-1β, TNF and IL-6 by macrophages infected for 24 hr, without or with 10 mM 2-DG, 30 μM 3PO and 500 nM 6-AN (n=6). (g) Gating strategy employed for FACS-based sorting of alveolar macrophages from naïve mice. (h) Levels of glucose determined in the blood of mice after 1 day of infection (n=9, representative of one out of three independent experiments). (i) Viability of CD11b^{+} and alveolar macrophages (%) in the lungs of 2-DG-treated mice after 1 day of infection with *A. fumigatus* (n=6) or following a mock challenge (PBS). Data are expressed as mean values ± SEM; P values were calculated using Student's two-tailed t test.

Supplementary Fig. 3

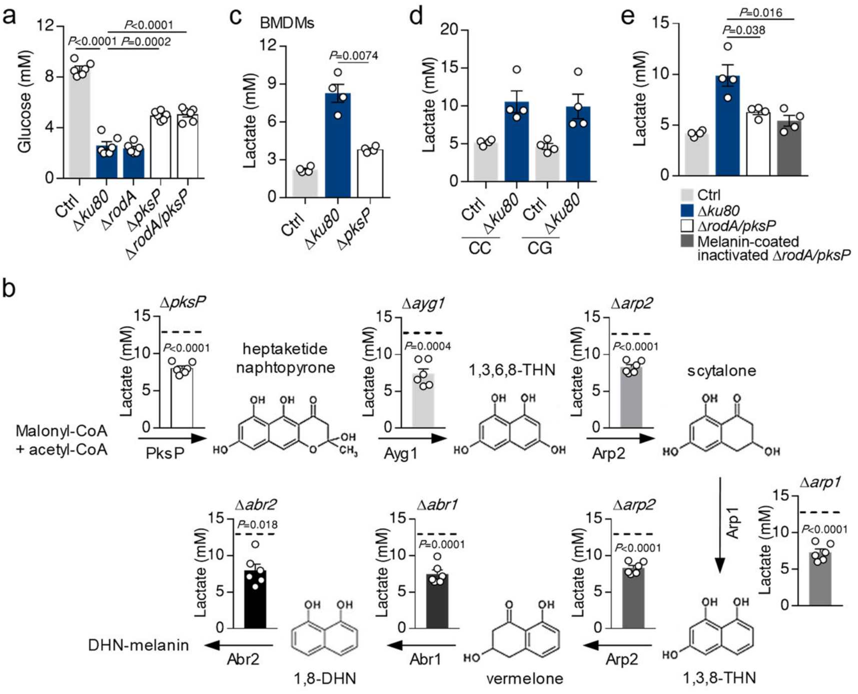

Supplementary Fig. 3 | Fungal melanin induces host glucose metabolism. (a) Glucose consumption by macrophages left untreated (Ctrl) or infected with the Δ*ku80*, Δ*rodA*, Δ*pksP* or Δ*rodA/pksP* strains of *A. fumigatus* for 24 hr (n=6). (b) Lactate secretion by macrophages infected with deletion mutants in the genes along the biosynthetic pathway of DHN-melanin for 24 hr (n=6). The dotted line represents the levels of secreted lactate determined upon infection with the B-5233 parental strain. The different compounds synthesized at each step along the biosynthetic pathways are illustrated. (c) Lactate secretion by BMDMs from C57BL/6 mice infected with the Δ*ku80* or Δ*pksP* strains for 24 hr (n=4). (d) Lactate secretion by macrophages isolated from carriers with distinct genotypes of the Gly26Ala SNP in MelLec and infected for 24 hr (n=4). (e) Lactate secretion by macrophages infected with live conidia of the Δ*ku80* or Δ*rodA/pksP* strains, or UV-inactivated conidia of the Δ*rodA/pksP* strain coated with 600 µg/mL of DHN-melanin for 24 hr (n=4). Data are expressed as mean values ± SEM; P values were calculated using Student's two-tailed t test.

Supplementary Fig. 4

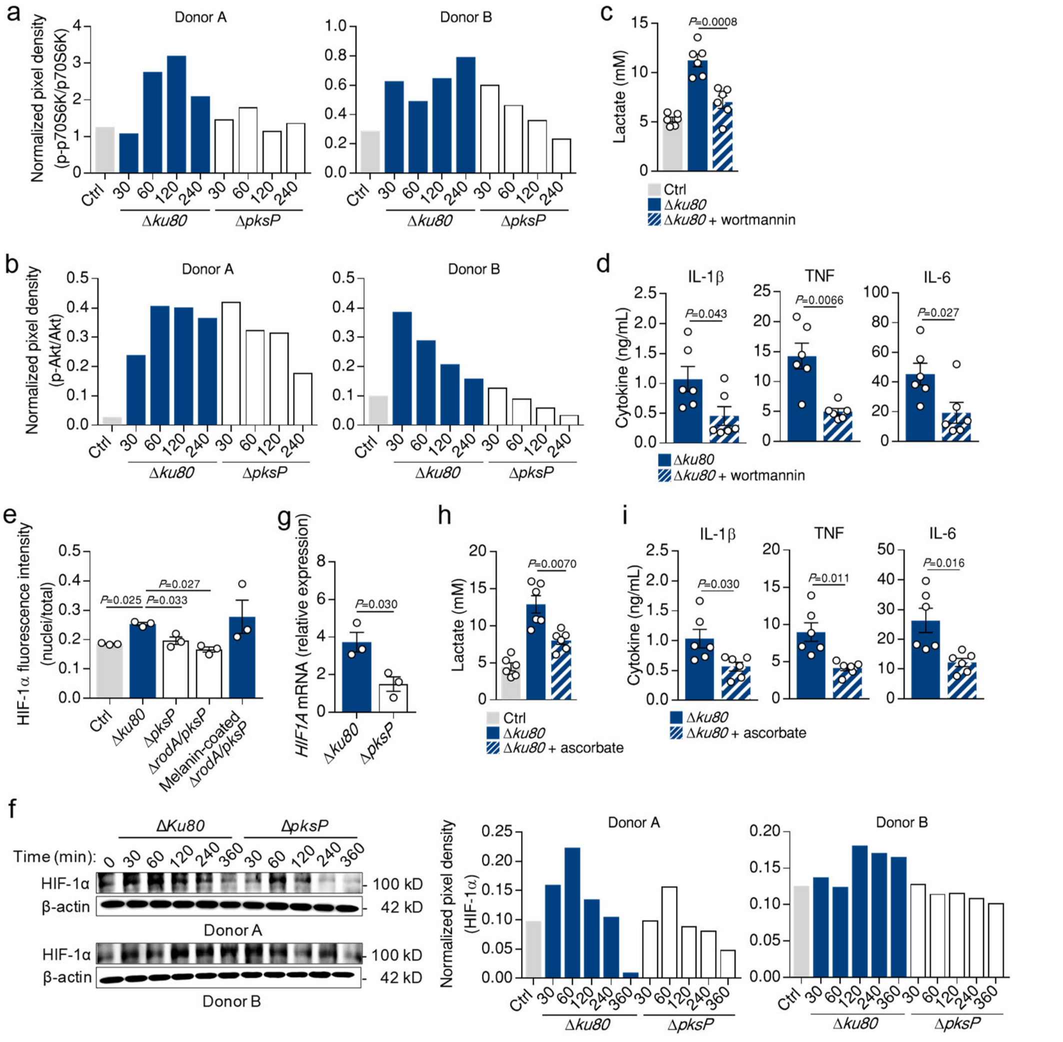

Supplementary Fig. 4 | *A. fumigatus* induces the metabolic shift of macrophages via mTOR and HIF-1α. (a) Densitometric analysis of the p-p70S6K/p70S6K ratio and (b) the p-Akt/Akt ratio, both normalized to β-actin, in macrophages infected with the Δ*ku80* or Δ*pksP* strains for 4 hr, with β-actin used as loading control (n=2). (c) Lactate secretion and (d) production of IL-1β, TNF and IL-6 by macrophages infected with the Δ*ku80* strain for 24 hr without or with 10 μM wortmannin (n=6). (e) Expression of HIF-1α in macrophages left untreated (Ctrl) or infected with the Δ*ku80*, Δ*rodA/pksP* or the melanin-coated Δ*rodA/pksP* strain for 2 hr (n=3). Results are expressed as fluorescence intensity (nuclear/total fluorescence) of at least 60 cells for each condition. (f) Levels of total HIF-1α in macrophages infected with the Δ*ku80* or Δ*pksP* strains for 6 hr. The densitometric analysis was performed by comparing the pixel density of the HIF-1α/β-actin ratio (n=2). (g) mRNA expression of *HIF1A* in macrophages infected with the Δ*ku80* or Δ*pksP* strains for 2 hr relative to uninfected cells (n=3). (h) Lactate secretion and (i) production of IL-1β, TNF and IL-6 by macrophages infected with the Δ*ku80* strain for 24 hr without or with 50 μM (+)-sodium L-ascorbate (n=6). Data are expressed as mean values ± SEM; P values were calculated using Student's two-tailed t test.

Supplementary Fig. 5

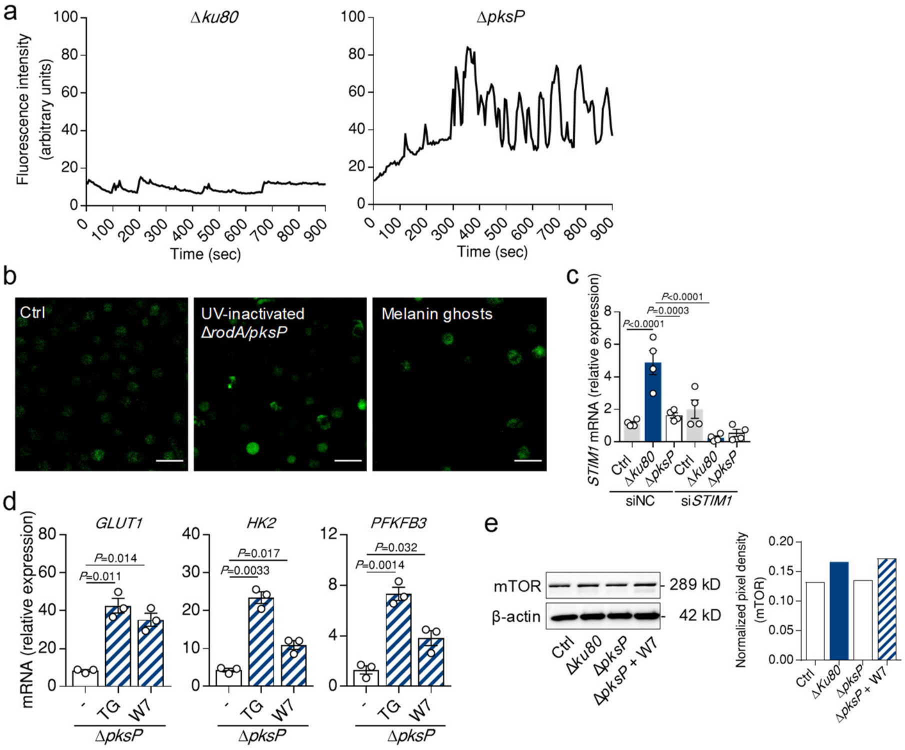

Supplementary Fig. 5 | Inhibition of calcium signaling enables host glucose metabolism in response to *A. fumigatus*. (a) Representative patterns of cytosolic calcium spikes in macrophages preloaded with the calcium indicator Fluo-4-AM and infected with the Δ*ku80* or Δ*pksP* strains. (b) Micrographs of macrophages preloaded with the calcium indicator Fluo-4-AM and left untreated (Ctrl) or stimulated with UV-inactivated Δ*rodA/pksP* conidia or melanin ghosts for 15 min (representative of three independent experiments). Scale bars, 35 µm. (c) mRNA expression of *STIM1* in macrophages left uninfected (Ctrl) or infected and silenced with a *STIM1* siRNA (si*STIM1*) (n=4). A scrambled siRNA was used as negative control (siNC). (d) mRNA expression of *GLUT1*, *HK2* and *PFKFB3* in macrophages infected with the Δ*pksP* strain for 2 hr relative to uninfected cells without or with 2 µM TG or 25 µM W7 (n=3). (e) Total mTOR in macrophages infected for 2 hr with the Δ*ku80*, Δ*pksP* or Δ*pksP* strains with 25 µM W7, with β-actin used as loading control (representative of two independent experiments). The pixel density of mTOR was normalized to β-actin. Data are expressed as mean values ± SEM; P values were calculated using Student's two-tailed t test or one-way ANOVA with Tukey's multiple comparisons test.

Supplementary Table 1. List of primers.

Primer name		Primer sequence (5´-3´)
hACTB	Forward	CTTCCAGCCTTCCTTCCTGG
	Reverse	ATTGCCAGGGTACATGGTGG
hGLUT1	Forward	ACTCATGACCATCGCGCTAG
	Reverse	GGACCCTGGCTGAAGAGTTC
hHK2	Forward	TTGACCAGGAGATTGACATGGG
	Reverse	CAACCGCATCAGGACCTCA
hPFKFB3	Forward	ATTGCGGTTTTCGATGCCAC
	Reverse	GCCACAACTGTAGGGTGGT
hHIF1A	Forward	GCTTTAACTTTGCTGGCCCC
	Reverse	TTTTCGTTGGGTGAGGGGAG
hSTIM1	Forward	GTCACAGTGAGAAGGCGACA
	Reverse	TGTGGATGTTACGGACTGCC
mUbb	Forward	TGGCTATTAATTATTCGGTCTGGAT
	Reverse	GCAAGTGGCTAGAGTGCAGAGTAA
mGlut1	Forward	CACTGTGGTGTCGCTGTTTG
	Reverse	AAAGATGGCCACGATGCTCA
mHk2	Forward	CACCCTACAGCAGCTGTGAA
	Reverse	TCTCCATCTCCACCCTCTGG
mPfkfb3	Forward	TGTCCAGCAGAGGCAAGAAG
	Reverse	TCTCGTTGAGTGCCTTCCAC
af18S	Forward	ATGGCCGTTCTTAGTTGGTG
	Reverse	GAGCCGATAGTCCCCCTAAG
afhxkA	Forward	TGACCACTTCGTCAAGGAGC
	Reverse	GGCAGATATCGAAGCCTCCC
afpfkA	Forward	TTGACTTCCTGCGGGAGAAC
	Reverse	CCTTGGCCTCCTCCTTGAAG
